# Feasibility of Using Elastic Wave Velocity Monitoring for Early Warning of Rainfall-Induced Slope Failure

**DOI:** 10.3390/s18040997

**Published:** 2018-03-27

**Authors:** Yulong Chen, Muhammad Irfan, Taro Uchimura, Ke Zhang

**Affiliations:** 1 State Key Laboratory of Hydroscience and Engineering, Tsinghua University, Beijing 100084, China; 2 Department of Civil Engineering, The University of Tokyo, Tokyo 113-8656, Japan; uchimura@civil.t.u-tokyo.ac.jp; 3 Department of Civil Engineering, University of Engineering & Technology Lahore, Lahore 54890, Pakistan; irfan@uet.edu.pk; 4 Faculty of Electric Power Engineering, Kunming University of Science and Technology, Yunnan 650500, China

**Keywords:** landslide, slope, monitoring, early warning, rainfall, elastic wave velocity

## Abstract

Rainfall-induced landslides are one of the most widespread slope instability phenomena posing a serious risk to public safety worldwide so that their temporal prediction is of great interest to establish effective warning systems. The objective of this study is to determine the effectiveness of elastic wave velocities in the surface layer of the slope in monitoring, prediction and early warning of landslide. The small-scale fixed and varied, and large-scale slope model tests were conducted. Analysis of the results has established that the elastic wave velocity continuously decreases in response of moisture content and deformation and there was a distinct surge in the decrease rate of wave velocity when failure was initiated. Based on the preliminary results of this analysis, the method using the change in elastic wave velocity proves superior for landslide early warning and suggests that a warning be issued at switch of wave velocity decrease rate.

## 1. Introduction

Landslides are one of the major natural disasters in mountainous regions. Rainfall is commonly known as a major landslide trigger in different geomorphological settings. Every year landslides initiated by intense or prolonged rainfall produce casualties and economic damages worldwide [1].

To reduce the damage caused by landslides, an early warning system is necessary to enable the early detection of landslide indicators and timely evacuation of residents from landslide-prone areas. So far real-time monitoring and early warning methods of landslides have been attempted, the most important of which are briefly by monitoring (1) actual slope movements through tilt sensors, inclinometers, extensometers, optical fiber sensor and so forth. [2,3,4,5,6,7]; (2) soil moisture variation using dielectric moisture sensors, tensiometers and so forth. [8,9,10], (3) rainfall record by rain gauge [11,12]; (4) acoustic emission with acoustic sensors [13,14,15,16]; and (5) electrical resistivity by electrodes [17,18].

However, monitoring of (1) slope movements and (2) soil moisture variation are limited to a small sensing area of the sensors. Such sensors are point sensors and hence, sensitive only to deformation and moisture changes in their own vicinity. To cover a wide potential landslide area, a large number of such sensors would be required. This could significantly increase the project cost and on the other hand, reducing the number of sensors would decrease efficiency of landslide prediction thereby making the technology itself questionable. A possible drawback of using (3) rainfall record is that the rainfall criterion does not take into account the effects of local geology, topography and human actions in a sloping ground. Moreover, the rainfall forecast cannot capture the local variation of rain in a small spatial scale such as hundreds of meters. In the case of (4) acoustic emission monitoring, the low-energy acoustic emission signals generated in soils attenuate significantly over short distances. The main disadvantages of (5) electrical resistivity measurement are the decrease of resolution with depth, the non-uniqueness of solutions for data inversion and interpretation and the in-direct information.

The confluence of these factors necessitates the exploration and development of new alternative reliable landslide monitoring and early warning techniques. An alternative method should consist in real-time monitoring for detecting and interpreting pre-failure soil deformations and be of the high spatial and temporal sensor resolution. In this paper, a new idea to monitor slope movements and soil moisture variations by using elastic wave propagation in soil has been presented. The aspire of this study was to evaluate the response of elastic wave velocities to moisture content and deformation of soil in the process of slope failure due to intensive rainfalls. A series of fixed and varied slope model tests, as well as a large-scale model test, were conducted. Fixed slope model tests were performed to understand the link between elastic wave velocity and soil water content and tilt. Varied slope model tests were performed to understand separately the influence of elastic wave velocity on soil water content and tilt. Large-scale model test was performed to validate the early warning system. The results of these tests were used to evaluate the potential of the elastic wave velocity monitoring as a prediction and early warning method of landslide. Based on the findings of the experimental program, expected operation of the elastic wave velocity monitoring system for landslide prediction in the field application is presented.

## 2. Materials and Methods

### 2.1. Materials

**Figure 1:**
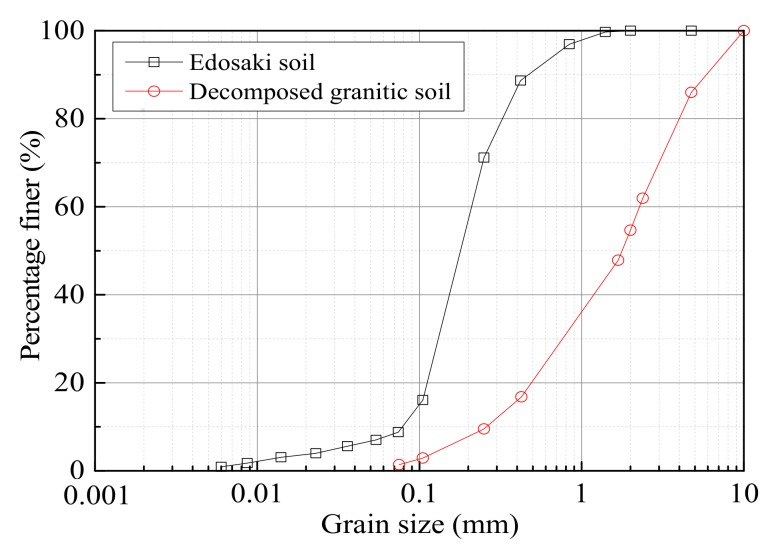
Grading curves of Edosaki and decomposed granitic soils.

Two soils, both from Japan, were used in this study: Edosaki soil (brown colored natural sand) procured from a trench pit in Ibaraki Prefecture for small-scale model tests and decomposed granitic soil retrieved from a natural slope in Hiroshima Prefecture for large-scale model test. Wet sieving analysis and hydrometer tests were performed on both the Edosaki and decomposed granitic soils as they exhibit fines (particles finer than 0.075 mm) contents of 9 and 1.4%, respectively. Figure 1 shows the grading curves of both materials. The basic soil properties measured in accordance with Japanese geotechnical society (JGS) standard test methods are listed in Table 1. Review of these results relative to the Unified Soil Classification System characterizes the Edosaki and decomposed granitic soils as well graded sand with silt (SW-SM) and poorly graded sand with gravel (SP), respectively.

### 2.2. Slope Model Tests

Laboratory model tests are considered as the most reliable method for studying the rainfall-triggered landslides. This is because the soil properties and boundary conditions can be controlled with the possibility of continuous monitoring of soil slope water content as well as subsequent deformation. For the purpose of better understanding the phenomenon related to elastic wave propagation in soil slope, during wetting and failure process as a result of rainfall, two types of small-scale slope model tests were performed in the Geotechnical Engineering Laboratory at the University of Tokyo. Fixed slope model tests were performed to understand the link between elastic wave velocity and soil water content and tilt, while varied slope model tests were carried out to understand separately the influence of elastic wave velocity on soil water content and tilt. Further, a large-scale slope model test was conducted to validate the early warning system at the National Research Institute for Earth Science and Disaster Prevention (NIED) in Japan.

#### 2.2.1. Small-Scale Fixed Slope Model Tests

**Figure 2:**
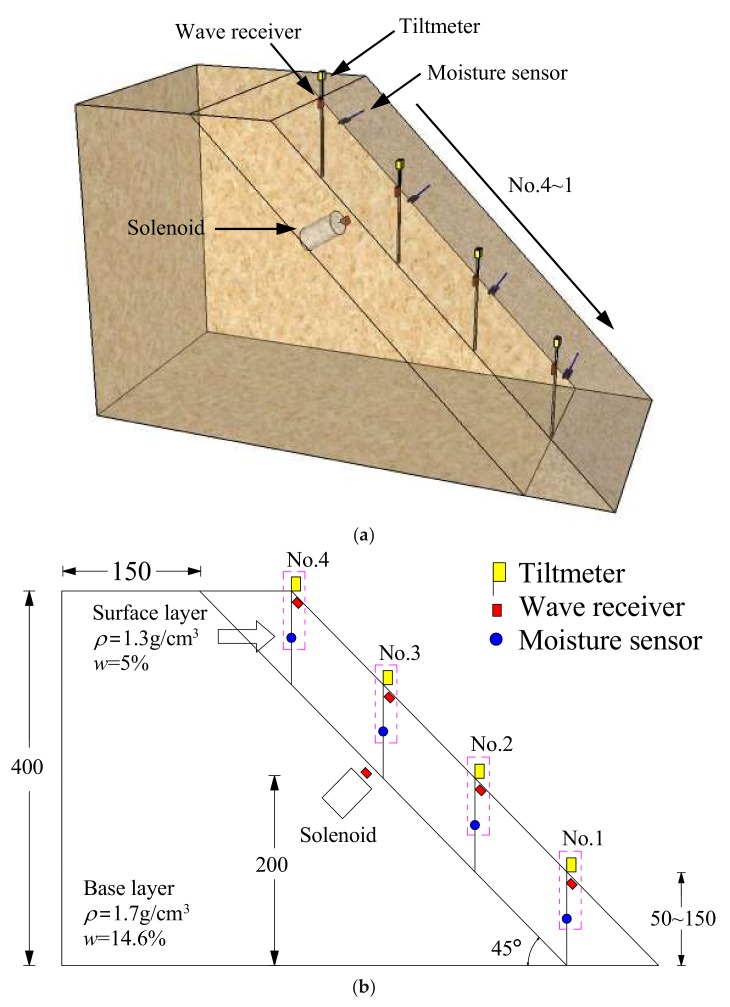
Schematic illustration of the fixed slope model. (**a**) 3-D illustration; (**b**) Scaled 2-D model (unit: mm).

The shape and size of slope model with the arrangement of measurement instrumentations are shown in Figure 2. The slope model was 40 cm high and 30 cm wide, with an inclination of 45°. Identical to the real slopes which have a loose/weathered surficial layer followed by a relatively dense/intact base layer, the model slope was prepared in two layers: a base layer and surface layer. The Edosaki sand in base layer was compacted to a dry density of 1.7 g/cm^3^ (degree of compaction (*D_c_*) = 96.5%) with optimum water content of 14.6% in the model container using an impact hammer. In contrast, the surface layer soil was loose with dry density of 1.3 g/cm^3^ (*D_c_* = 73.8%) and initial water content of 5%. Three different surface layer thicknesses of 5, 10 and 15 cm in the vertical were considered.

Soil moisture sensors (EC-5 by Decagon Devices, Inc., Washington, DC, USA) and tiltmeters based on Micro Electro Mechanical Systems (MEMS) technology [19,20] were used to measure volumetric water content and tilt angle in the unstable surface layer of slope. Soil moisture sensors and tiltmeters were connected to a data logger (HOBO RX3000 by Onset Computer Corporation, Bourne, MA, USA) for continuous recording of data. It was set to log data for every 1s from all the sensors connected with this logger. Four tiltmeters and moisture sensors were arranged on the slope to monitor the tilt angle and volumetric water content, respectively.

**Figure 3:**
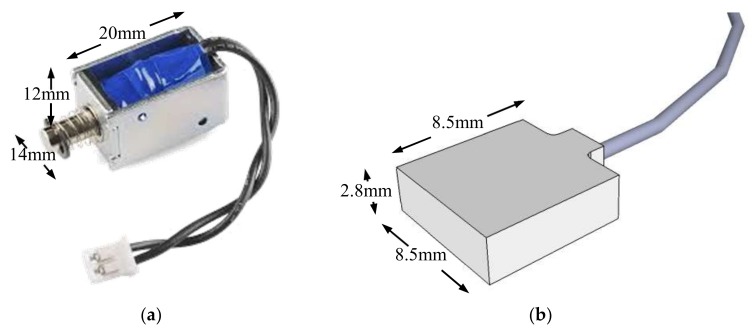
Solenoid and receiver used in small-scale model tests. (**a**) Solenoid; (**b**) Receiver.

**Figure 4:**
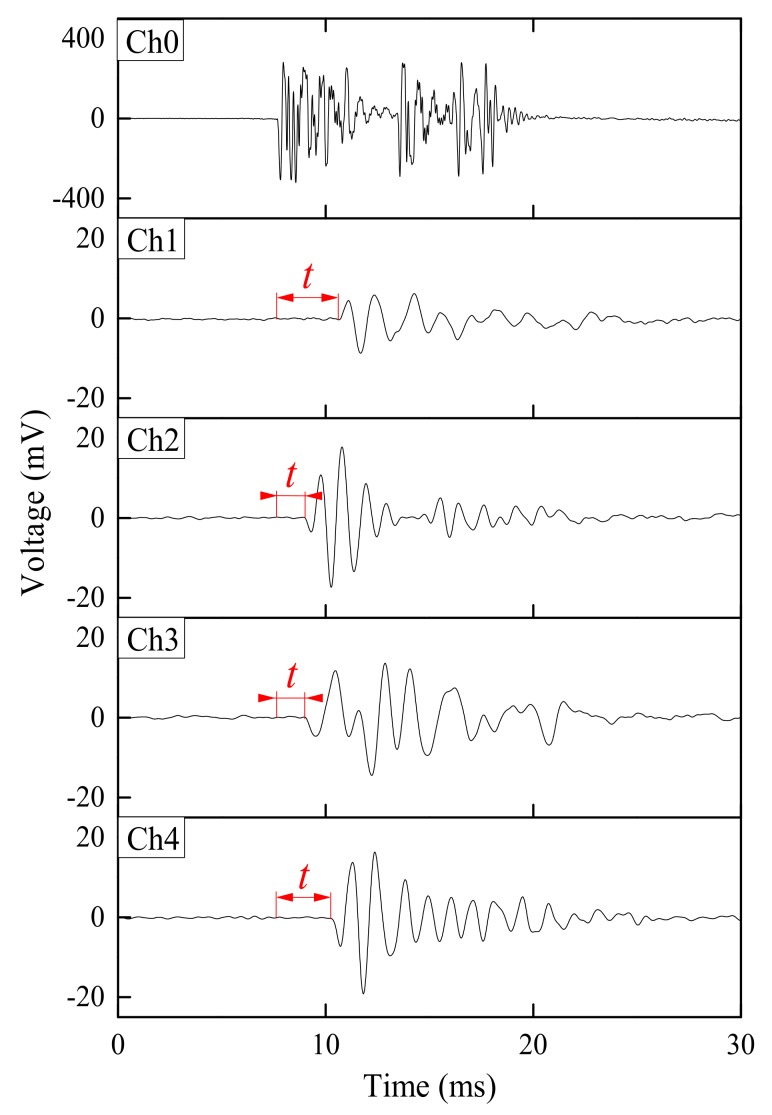
Example of transmitted and received signals obtained from small-scale model test.

The elastic wave monitoring system consisted of exciter, receiver, microcontroller and data acquisition unit. For small-scale model tests, a solenoid (ZHO-1040L/S by Zonhen Electric Appliances HK Co, Ltd., Guangdong, China, see Figure 3a), piezoelectric vibration sensors (VS-BV201 by NEC TOKIN Corporation, Sendai, Japan, see Figure 3b) and a data logger (NR-500 by Keyence Corporation Ltd., Osaka, Japan) were used to serve as wave exciter, receiver and data acquisition unit. A small plastic cylinder box housing solenoid was buried into the shallow surface of the base layer. The direction of movement of plunger as well as box was kept perpendicular to the slope. One of the wave sensors, that is, Ch0, was fixed adjacent to the solenoid, while the other four (Ch1–4) were installed at superficies of the slope to measure the wave travel time. A microcontroller (MSP430 by Texas Instruments, Dallas, TX, USA) with electronic accessories including resistors, capacitor, diode and power transistor were fabricated to generate twenty repeated pulses at a single electric input in a very short span of time. Twenty signals were stacked to increase the signal-to-noise ratio of the recorded waveforms. The travel time was estimated from the first arrival of the transmitted and received signals. A typical waveform of transmitted and received signals is shown in Figure 4. The elastic wave velocity was calculated using the travel distance and the travel time of the wave. In this study, we assumed that the initial distances between the source and each receiver measured along the profile in Auto Computer Aided Design (AutoCAD) were constant during the tests, although the locations of wave receivers were varied with soil deformations as a result of rainfall. Chen et al. [19] compared the elastic wave velocities in slope model tests with those from triaxial tests and revealed the wave velocity recorded in slope model tests to be S-type in nature.

Artificial rainfall was produced by spray nozzle (SSXP series by H. IKEUCHI & Co., Ltd., Osaka, Japan) installed above the model which provided uniform rainfall with intensity of 100 mm/h. The rainfall was continued until landslide initiated.

#### 2.3.2. Small-Scale Varied Slope Model Tests

**Figure 5:**
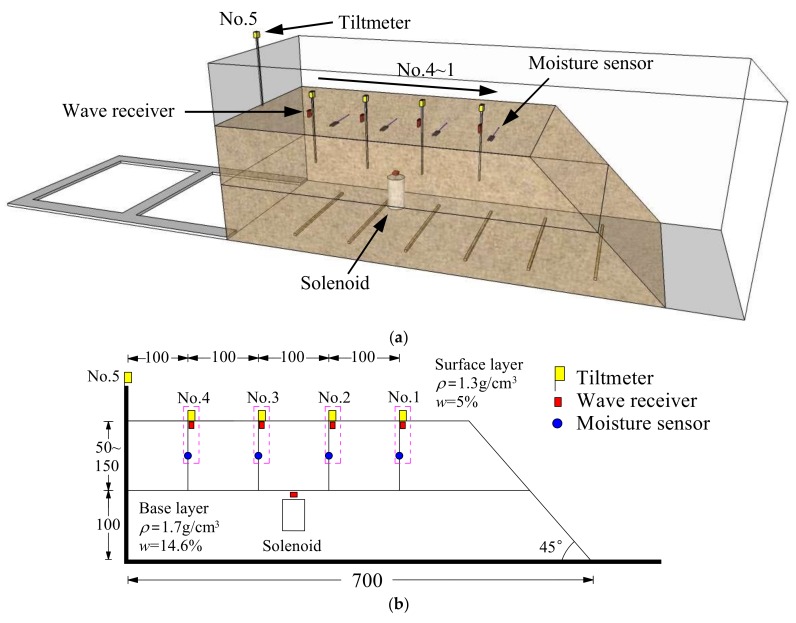
Schematic illustration of the varied slope model. (**a**) 3-D illustration; (**b**) Scaled 2-D model (unit: mm).

This type of model tests was carried out to segregate the effects of water content and deformation on the wave velocity. For this purpose, the authors designed a model container as illustrated in the Figure 5. The bottom was made to reproduce a frictional contact to prevent soil sliding by gluing wooden strips to it. This slope model was featured by a long slope crest and was 30 cm in width and 70 cm in length, with slope angle of 45°. The compacted soil slope consisted of two layers; base layer (*ρ_d_* = 1.7 g/cm^3^, *w*_initial_ = 14.6%) and surface layer (*ρ_d_* = 1.3 g/cm^3^, *w*_initial_ = 5%). Surface soil layer was 5, 10 or 15 cm in vertical thickness for different tests performed. The box housing solenoid was buried close to the surface of base layer. Five wave receivers, four tiltmeters and moisture sensors were installed on the slope.

The side-walls of the container were made of wooden plates on which the friction angle was found to be 14°, whereas the friction angle on the interface between the dense and loose layers was found to be 45°. Hence the friction to support the soils in upper layer was mainly provided by the dense soil layer, rather than side walls. The effect of side wall friction was further explored by conducting tests with model widths ranging from 5 cm to 35 cm; and it was found that the effect of side wall friction could be ignored for slope with width larger than 15 cm [21]. The 30 cm wide model used in this study was therefore considered free from boundary effects.

Two stages of test scheme were applied. During the first stage, rainfall was continuously applied for 10 min. In this stage, only water content was changed, with little variation in shear deformation of soils. Hence, the variation of wave velocity in the rainfall stage completely resulted from the increase of water content. In the second stage, rainfall was stopped and the model was raised slowly by chain block until its inclination became 40°. No failure was observed due to such tilting. Shear deformation in the samples is designated in terms of relative inclination which is calculated as the difference between tilt angles monitored by the tiltmeters No. 1~4 installed in soil and the sensor No. 5 attached to the model container. Shear deformation thus increased with model inclination under constant water content. That is, the change of wave velocity in the inclined stage was totally attributed to the increase of inclination (i.e., shear deformation). Wave signal was recorded during both test stages. 

#### 2.3.3. Large-Scale Slope Model Test

**Figure 6:**
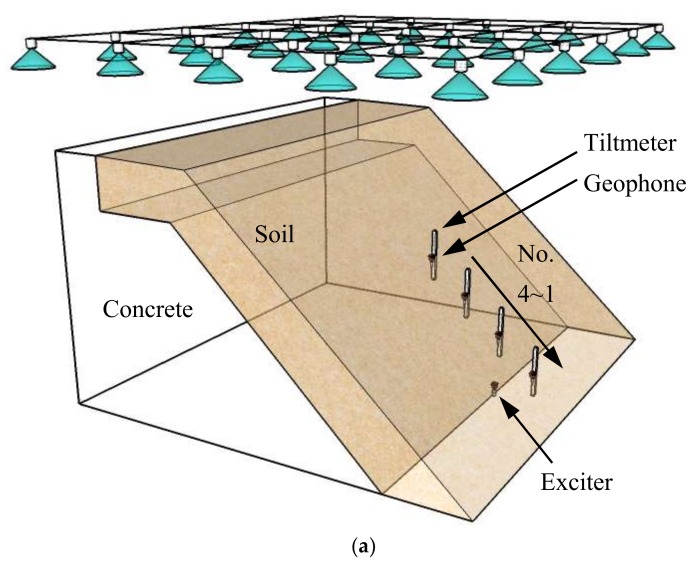

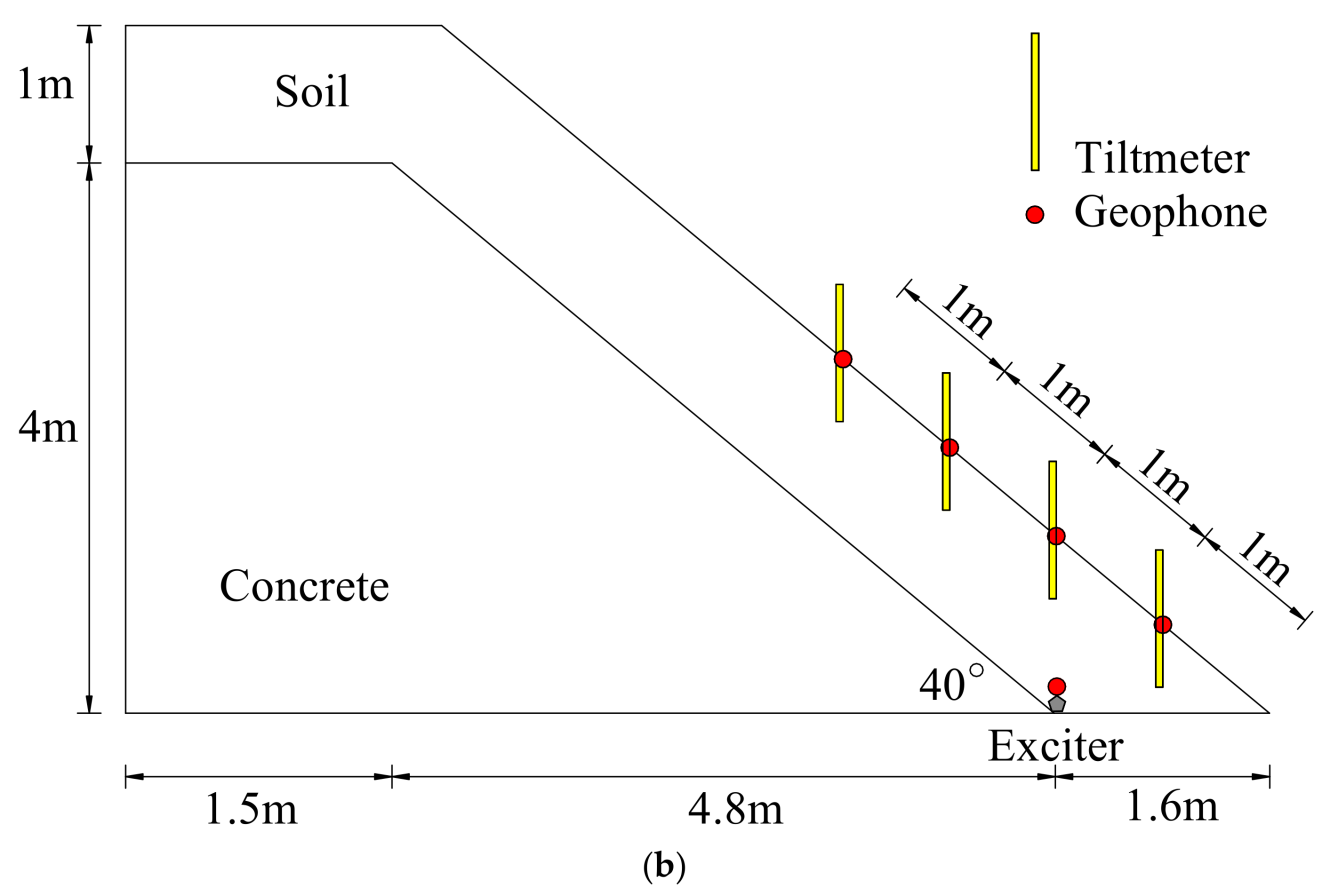
Schematic illustration of the large-scale slope model. (**a**) 3-D illustration; (**b**) Scaled 2-D model.

**Figure 7:**
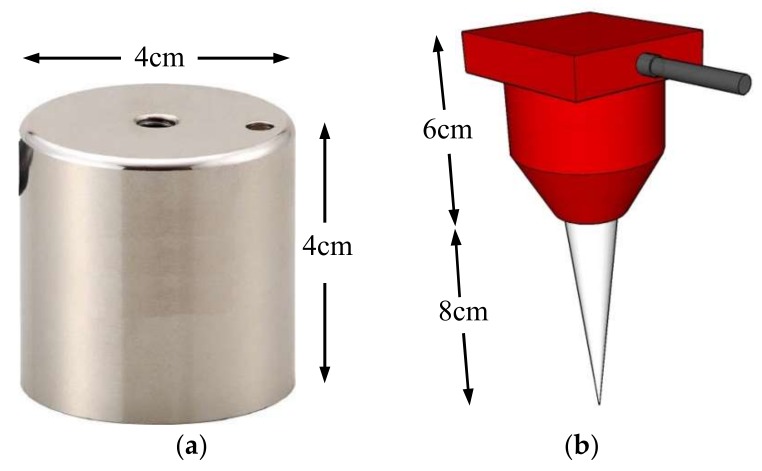
Electromagnetic holder and geophone used in large-scale model tests. (**a**) Electromagnetic holder. Similar to the solenoid, when an electric current is given, the electromagnetic holder produces magnetic field to attract an iron nut (*m* = 92.6 g) upwards to hit itself. As a result, elastic wave is created. The nut falls down due to gravity upon removal of electric current; (**b**) Geophone.

**Figure 8:**
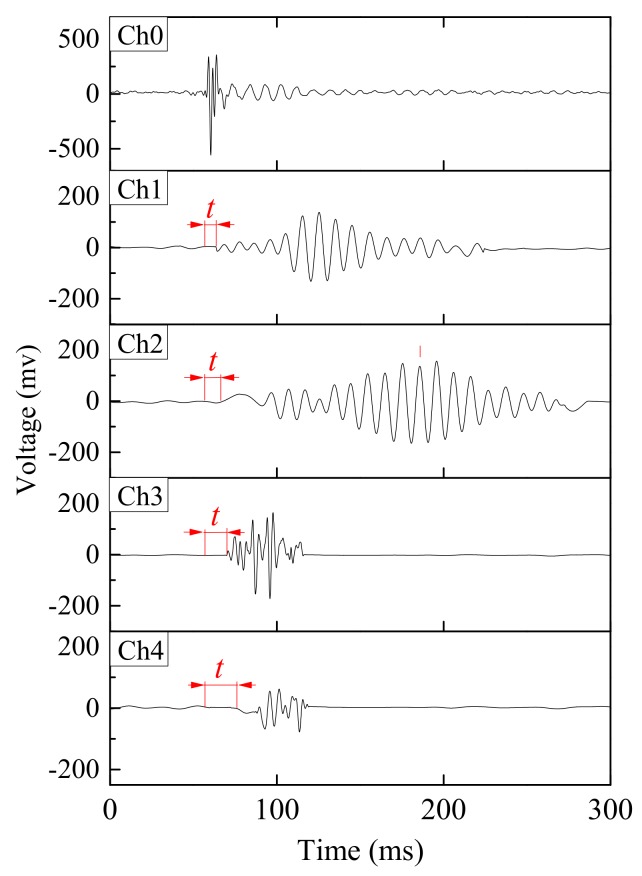
Example of transmitted and received signals obtained from large-scale model test.

The large-scale slope model was prepared on a concrete base and its geometry is shown in Figure 6. The slope model was 7.9 m long, 5 m high and 9 m wide and the slope angle was 40°. The monitoring system consisted of tiltmeters and the wave sensors, to monitor the variable of interest. A robust electromagnetic holder (TMEH-A4 by Trusco Nakayama Corporation, Tokyo, Japan, see Figure 7a), geophones (GS-11D by OYO Geospace Corporation, Annecy, France, see Figure 7b) and a data recorder (ZR-MDR10 by OMRON Industrial Automation, Kyoto, Japan) were used to serve as wave exciter, receiver and data acquisition unit. The microcontroller was employed to reduce the noise and the result with stacking of 20 pulses is presented in Figure 8.

Instrumentation was done close to the toe of slope, since the toe is expected to accumulate moisture and initiate failure first [22]. A box housing the electromagnetic holder was buried close to the toe of the concrete base. Five wave sensors, one placed next to the exciter at the toe of the concrete base and four installed at superficies of surface layer, were applied to measure the elastic wave velocity. Four tiltmeters measuring the tilt angle of a wooden rod installed to a depth of 0.5 m in the unstable soil layer on the slope surface were employed. The tilt angle and elastic wave velocity were recorded in real-time (the former every 1 min and the latter every 5 min). Rainfall simulator was used to realize uniform rainfall during the model tests. The rainfall simulator was mounted above the slope model and was capable to produce rainfall intensities up to 300 mm/h in the form of tiny raindrops in order to create a uniform distribution of rainfall. Water was pumped to the nozzles using a constant-rate pump. The rainfall intensity is controlled by the opening of nozzles and the applied water pressure. In the present study, an artificial continuous rainfall of 70 mm/h was produced. 

## 3. Results

### 3.1. Small-Scale Fixed Slope Model Tests

**Figure 9:**
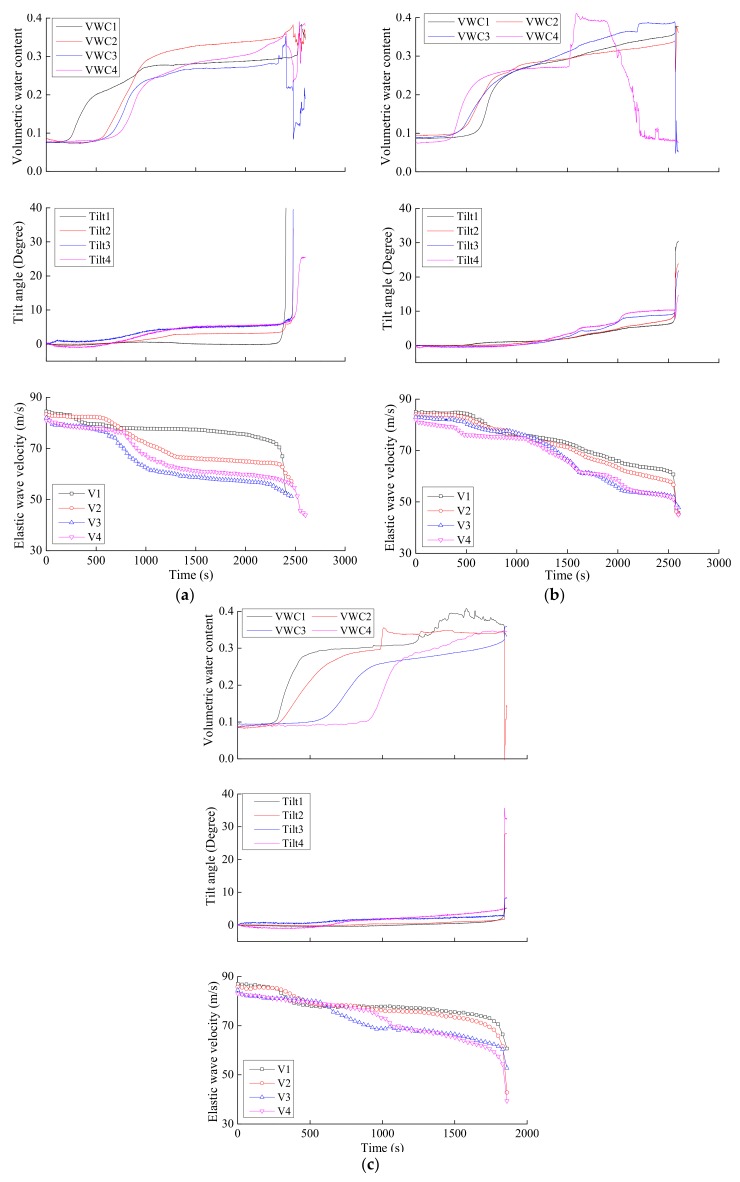
Time series data of volumetric water content, tilt angle and elastic wave velocity by fixed model tests with surface layer thickness of (**a**) 5 cm, (**b**) 10 cm and (**c**) 15 cm.

Figure 9 gives the time histories of volumetric water content, tilt angle and elastic wave velocity from the onset of rainfall during fixed slope model tests. As the rainfall progressed, the sensors located in the lower part of slope showed an increase in volumetric water content, forming a water table below the toe area of the slope, leading to a progressive saturation or near saturation of the soil mass in the upper part of the slope. Very small tilt deformation occurred before the collapse of the slope and the collapse itself suddenly occurred, resulting in a sharp increase in tilt angle.

In response to increasing volumetric water content and tilt deformation of soils, elastic wave velocity decreased gradually, followed by a large drop as slope instability initiated. In early stage, there was an increase just in the volumetric water content with no change in tilt angle of soil. Thus, it is deduced that the slight decrease in elastic wave velocity in early stage was caused by increase in volumetric water content. While in the latter stage, the volumetric water content remained at a high level with no significant increase but tilt angle started to increase. Therefore, the apparent decrease in elastic wave velocity in the latter stage was thought to result from the increase in tilt deformation. Unfortunately, the elastic wave velocity data ended prior to slope failure because of its sudden collapse and the sampling interval of elastic waves set as 30 s. The final significant drop in wave velocity was thus not available. However, based on the monitoring of trends from recorded data, it was expected that the elastic wave velocity would reduce largely as the slope failure occurred.

**Figure 10:**
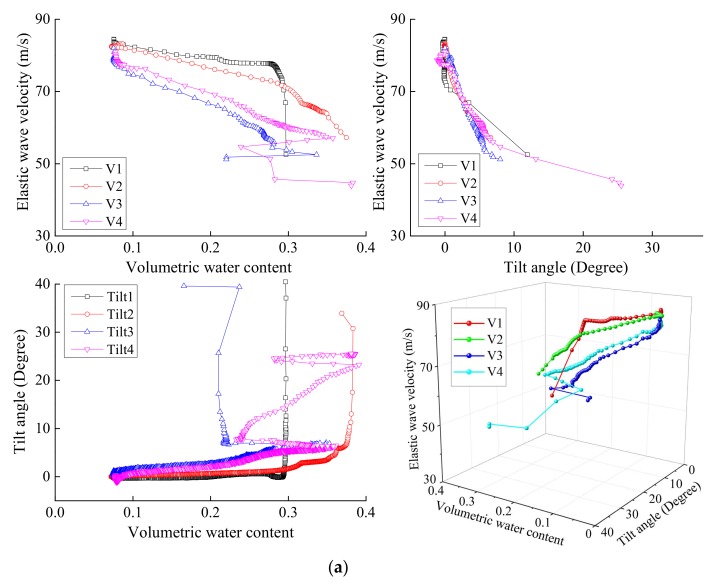

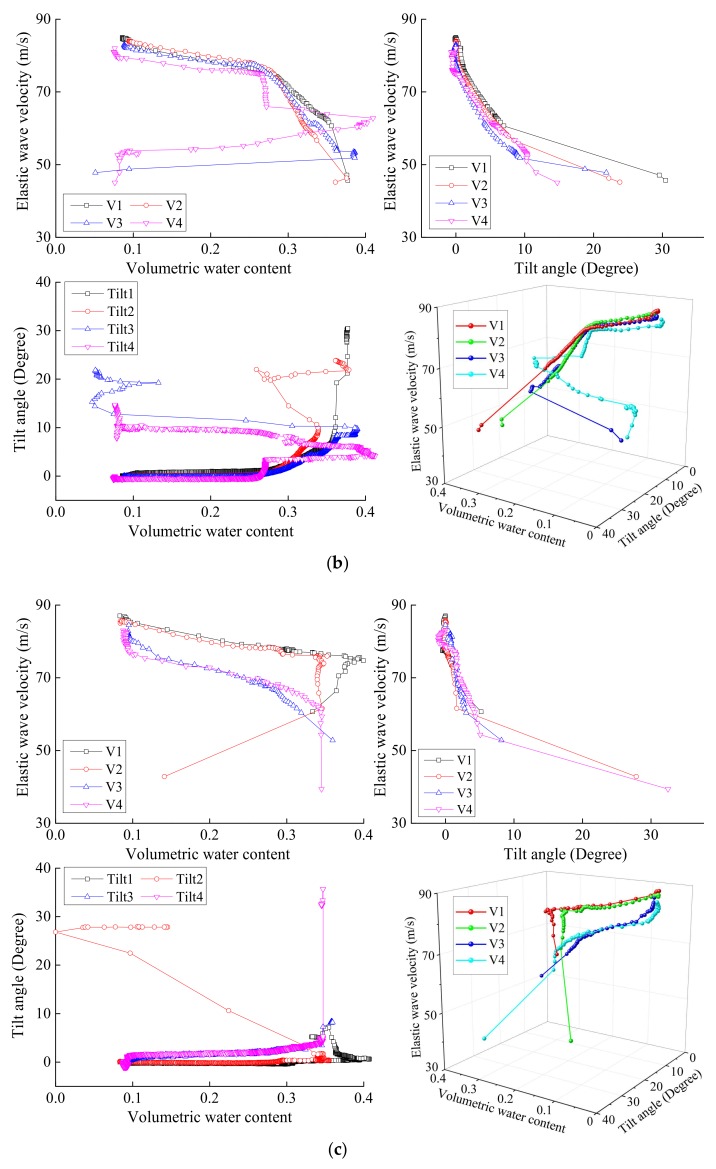
Relationship between elastic wave velocity against volumetric water content and tilt angle by fixed model tests with surface layer thickness of (**a**) 5 cm, (**b**) 10 cm and (**c**) 15 cm.

Figure 10 shows the relationship between tilt angle, volumetric water content and the elastic wave velocity during fixed model tests. The elastic wave velocity-volumetric water content-tilt angle curves showed that the elastic wave velocities decreased gradually in the beginning and then decreased rapidly due to tilt deformation. While the initial decrease in elastic wave velocity was without any significant increase of tilt angle (i.e., as a result of volumetric water content increase). The subsequent sharp decrease owed to tilt deformation. The three-dimensional plots of relationships between elastic wave velocity against volumetric water content and tilt angle showed that a gradual decrease in elastic wave velocities was followed by a rapid decrease once the failure was initiated. Wave velocity continued decreasing with an accelerated rate by the coupled effect of increasing water content and deformation that appeared to be interrelated.

### 3.2. Small-Scale Varied Slope Model Tests

**Figure 11:**
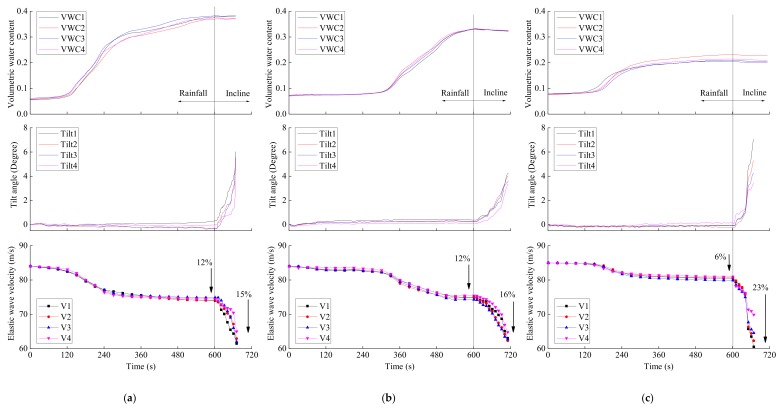
Time series data of volumetric water content, tilt angle and elastic wave velocity by varied model tests with surface layer thickness of (**a**) 5 cm, (**b**) 10 cm and (**c**) 15 cm.

**Figure 12:**
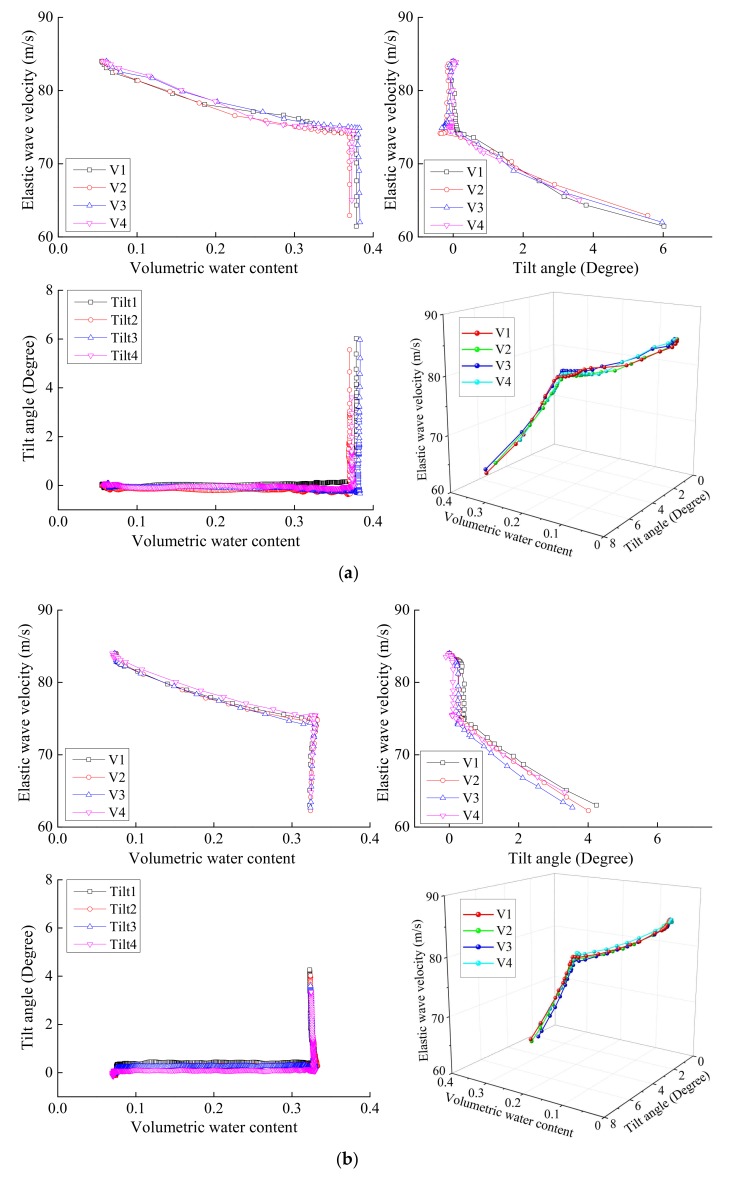

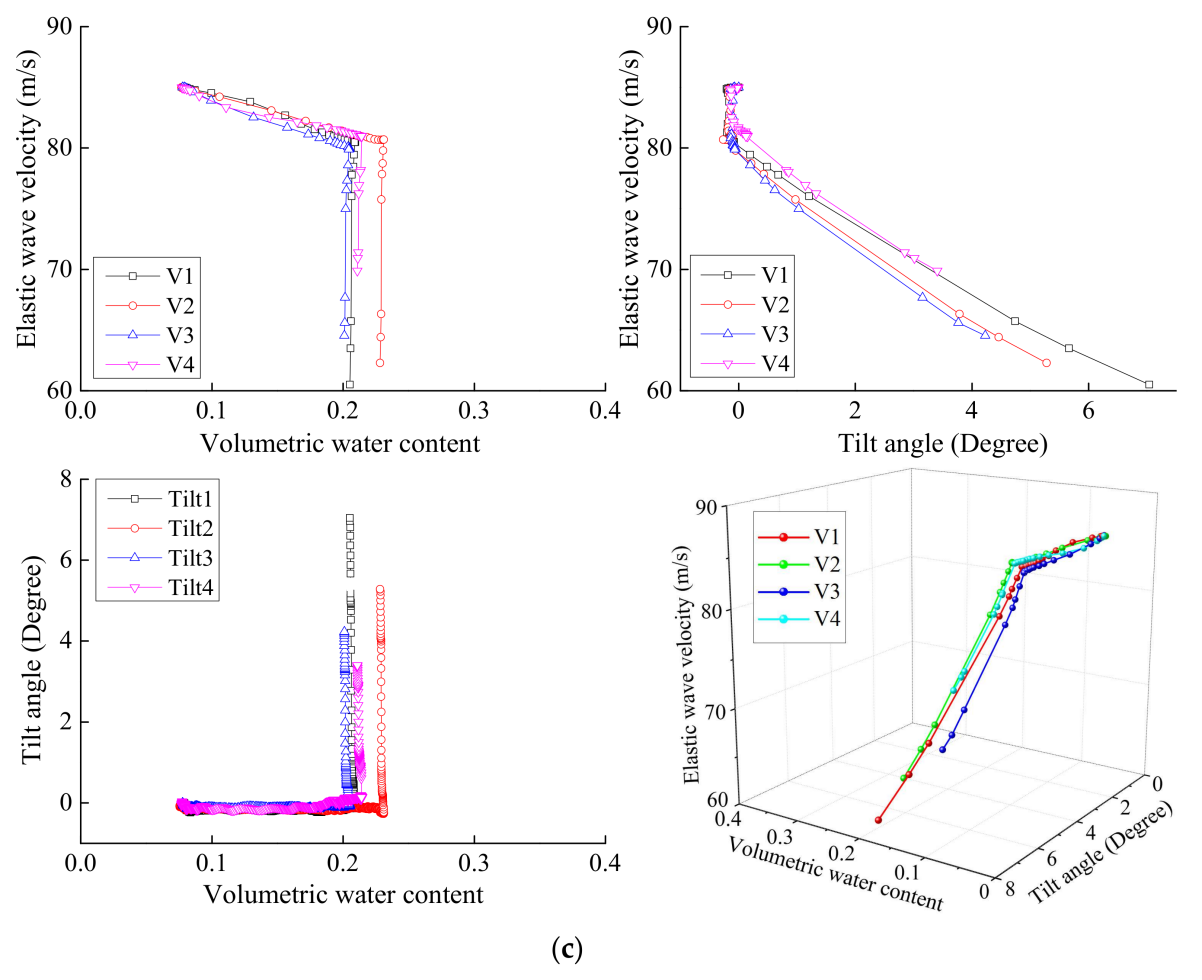
Relationship between elastic wave velocity against volumetric water content and tilt angle by varied model tests with surface layer thickness of (**a**) 5 cm, (**b**) 10 cm and (**c**) 15 cm.

The volumetric water content, tilt angle and elastic wave velocity monitored during varied model tests for slope with 5~15 cm in surface layer thickness are plotted in Figure 11. During rainfall stage, moisture infiltrated into the surface thereby increasing the volumetric water content. Meanwhile, only small increase in tilt angle was observed. During this phase, the elastic wave velocity decreased by around 12% when surface layer thickness was 5 and 10 cm and by 6% when surface layer thickness was 15 cm. The volumetric water content increase for 5 and 10 cm surface layer thickness was nearly the same (Figure 11a,b). The corresponding reduction in elastic wave velocities was also similar. For 15 cm surface layer thickness, increase in volumetric water content was significantly lower than other two cases. The corresponding decrease in elastic wave velocities was also much less than other two cases. Such observation supports the results of Irfan et al. [23] who showed that the decrease in wave velocities in partially saturated specimens is proportional to increase in moisture. Such decrease in wave velocities during initial phase of these model tests can be attributed to the change in moisture content only since negligible tilting was observed during this phase. After 10 min from the start of experiment, rainfall was stopped and the model was lifted to an inclination of 40°. During such inclined stage, moisture content remained essentially constant whereas the tilt angles increased rapidly. When the surface layer thickness was 5, 10 and 15 cm, the elastic wave velocity decreased by around 15, 16, 23%, respectively, during this stage and this decrease could essentially be attributed to shear deformations only. This indicates that shear deformation had more significant influence on elastic wave velocities than water content. Figure 12 shows the relationship between the tilt angle, the volumetric water content and the elastic wave velocity during rainfall tests. The elastic wave velocity recorded by each sensor placed at different locations decreased along the same path with the increase in volumetric water content and tilt angle. It indicated that decreasing in elastic wave velocity depended slightly on water content and strongly on inclination.

### 3.3. Large-Scale Slope Model Test

**Figure 13:**
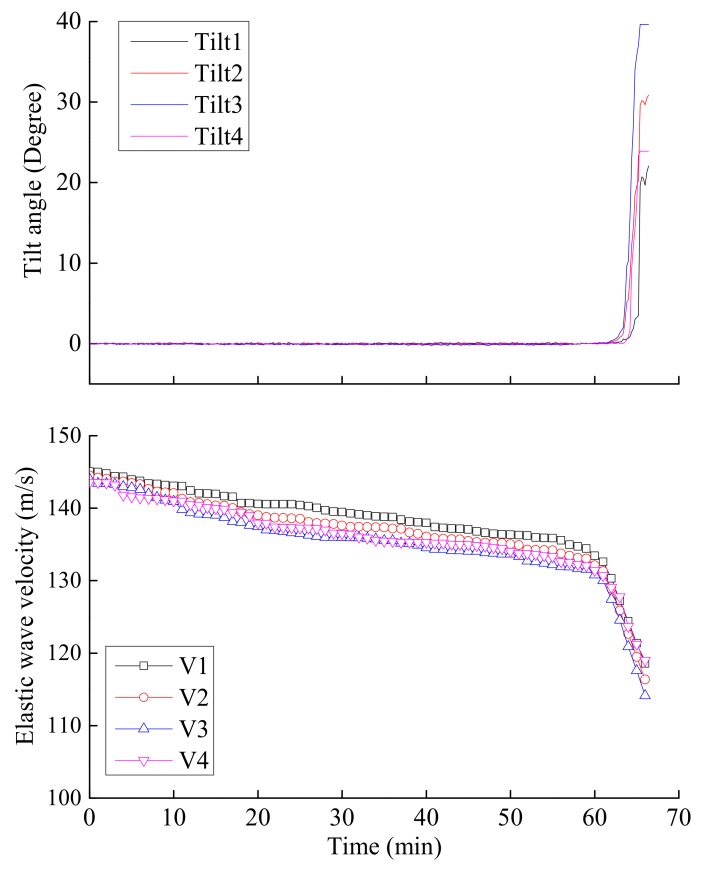
Time series data of tilt angle and elastic wave velocity obtained from the large-scale slope model test.

Figure 13 indicates the variations in tilt angle and elastic wave velocity at the different locations of the slope during rainfall event. The observed behavior is a bilinear one, showing two distinct stages, that is, changing from a steady state to a rapidly changing state as the rainfall progressed. Tilt angle of the slope started to increase when its toe was saturated at about 60 min after the beginning of rainfall. Prior to increase of tilt angle, the increase in moisture content only resulted in a small decrease in elastic wave velocity. However, once the onset of surface soil tilting was reached, elastic wave velocity decreased significantly. Thus, the observed behavior of large-scale slope model test is in agreement with the conclusions drawn from laboratory element tests by Irfan et al. [23], as well as small-scale model tests presented hereinbefore.

## 4. Velocity Measurement Errors Induced by the Variable Transmitter-Receiver Distances

The transducer movement may introduce significant measurement errors [24]. In other words, the measured wave velocity variations were partially due to the movement of wave receivers on the slope. Such artefacts due to the displacement of the receivers along the profile comprise a potential source of velocity measurement errors (due to distance errors). Here a discussion about the measurements of the distances between the source and the receivers, and the impact on the velocity errors was made.

**Figure 14:**
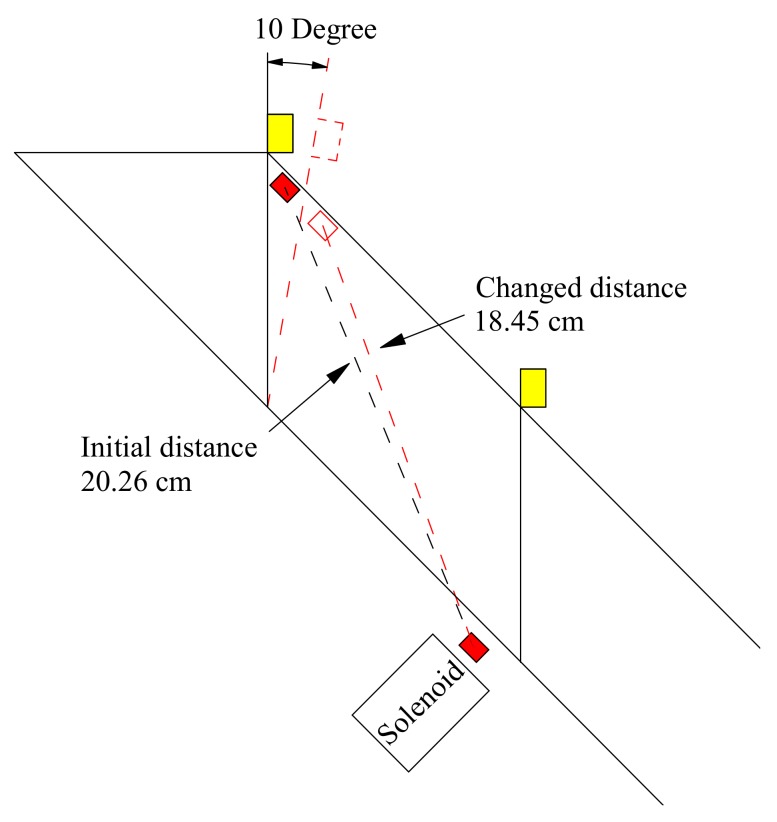
Schematic illustration of the movement of wave receivers and changed distances.

From small-scaled fixed model tests, the maximum tilt angle prior to slope failure was observed to be 10° at sensor No. 4 located near the top of slope for model having surface layer thickness of 10 cm, which was larger than those recorded in the small-scaled varied and large-scale model tests. The schematic illustration of the movement of wave receivers and changed distances between the transmitter and the receivers is given in Figure 14. In this case, when tilt deformation increased to 10°, the distance between the transmitter and the receivers was decreased from 20.26 cm to 18.45 cm, resulting in the distance error up to 8.93%. Compared to the decease by 48% in the elastic wave velocity, the distance error was too small to impact the decreasing tendency of wave velocity.

## 5. Application of Elastic Wave Velocity Monitoring for Landslide Prediction

**Figure 15:**
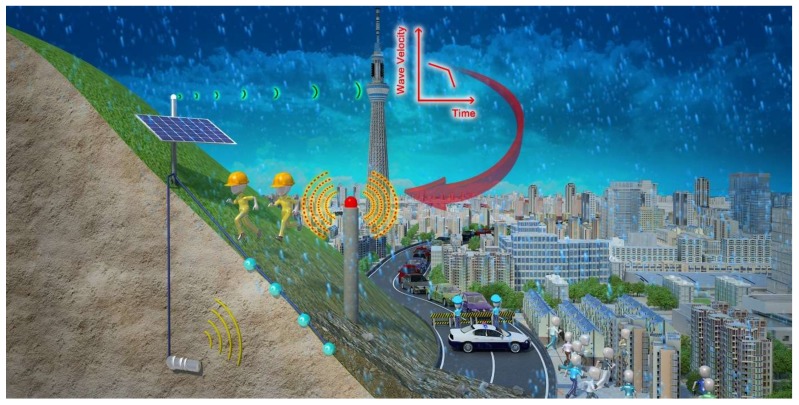
The operation of the elastic wave velocity monitoring system.

Experimental results presented in preceding sections lay down the basic framework and fundamental guidelines for the use of elastic wave velocities for monitoring the stability of slope. The technique is attractive because elastic wave velocity is strongly dependent on water saturation and deformation which are both key factors in slope stability. Slopes may be monitored over time to observe changes in the elastic wave velocities using an automated real-time monitoring system to collect and analyze data on a daily basis. The operation of the monitoring system based on detecting and quantifying elastic wave velocity through soil slope surface is illustrated in Figure 15. The monitoring system shall consist of a single exciter, several receivers, a data server and communication unit. A single exciter shall be installed in a borehole that penetrates any shearing surface or potential shear surface beneath the slope. Elastic waves generated by the exciter can be detected by the receivers at the ground surface. The wave signals can be transferred to data server by wireless communication and the corresponding wave velocities in response to moisture variations and soil deformation are derived by it. The data server can collect data from all the receivers. Thus, the abnormal behavior of slope can be detected as a precursor of failure; then a warning is issued. The exciter and receivers, as well as the data server can be powered by a battery which can be recharged by a solar panel so that the monitoring system can work semi-permanently without having to worry about the batteries. Elastic wave velocities can be automatically detected, recorded and time stamped at regular intervals of the order of minutes. As part of the data processing, an analog filter can be applied to eliminate environmental noise (such as that generated by wind, traffic and construction activities) and to focus wave signal detection. This can produce a robust system and minimize the potential for false warnings.

If a receiver detects a change in rate of decrease in wave velocity, the data server shall transfer this information to the communication unit through a wireless network link. The communication unit subsequently will send a warning to responsible institutes so that urgent actions can be taken (e.g., send an engineer to inspect and stabilize the slope, or immediately stop traffic and evacuate vulnerable people and so forth.).

## 6. Conclusions and Recommendations

Rainfall-induced landslides are among the most widespread hazards in the world. The demand for new reliable methods for landslide monitoring and early warning continues to increase and thereby reducing the damage risk to the human settlement, structures and environment over the landslides’ mass. This study was performed to determine a method for landslide early warning by real-time monitoring of elastic wave velocity in soil. A thorough laboratory investigation was conducted to explore the validity of this idea. All the above-mentioned results have consistently demonstrated that the elastic wave velocity was feasible to provide real-time information that could be used to assess the stability of soil slopes and give an early warning.

Changes in elastic wave velocity were well correlated with volumetric water content and slope deformation. Elastic wave velocity decreased gradually with moisture content of soil in pre-failure, while it decreased rapidly once the failure was initiated. Such sharp decrease in wave velocities upon failure initiation can be used to identify the failure initiation in a soil slope. Therefore, it is suggested that a warning be issued at the acceleration of decrease in elastic wave velocity.

In present study, the sensitivity of elastic wave velocities to soil saturation as well as soil deformations was well explored by means of model tests in controlled laboratory environment. However, practical application of these concepts for actual landslide early warning requires further confirmations in actual field conditions where several unforeseen parameters may also play their part. Artificial rainfall experiments on actual landslide sites may help in establishing a more realistic and practical approach for landslide early warning.

Measurement of elastic wave velocities in the field would require sophisticated transmitter and receiver devices. Cross-disciplinary research, bringing together the knowledge of electrical engineering and geotechnical engineering, is therefore required to develop efficient, low energy, low noise transmitter and receiver devices for landslide early warning systems.

## Figures and Tables

**Table 1:** Physical properties of Edosaki and decomposed granitic soils.

Properties	Edosaki Soil	Decomposed Granitic Soil
Specific gravity, *G_s_*	2.64	2.70
Mean grain size, *D*_50_ (mm)	0.18	1.78
Coefficient of uniformity, *C_u_*	2.82	8.78
Coefficient of gradation, *C_c_*	1.16	0.581
Maximum void ratio, *e*_max_	1.16	0.96
Minimum void ratio, *e*_min_	0.647	0.52

## References

[1] Salvati P., Bianchi C., Rossi M., Guzzetti F. (2010). Societal landslide and flood risk in Italy. Nat. Hazards Earth Syst. Sci..

[2] Uchimura T., Towhata I., Anh T.T.L., Fukuda J., Bautista C.J.B., Wang L., Seko I., Uchida T., Matsuoka A., Ito Y. (2010). Simple monitoring method for precaution of landslides watching tilting and water contents on slopes surface. Landslides.

[3] Uchimura T., Towhata I., Wang L., Nishie S., Yamaguchi H., Seko I., Qiao J. (2015). Precaution and early warning of surface failure of slopes using tilt sensors. Soils Found..

[4] Yang Z.J., Shao W., Qiao J.P., Huang D., Tian H.L., Lei X.Q., Uchimura T. (2017). A multi-source early warning system of MEMS based wireless monitoring for rainfall-induced landslides. Appl. Sci..

[5] Greif V., Sassa K., Fukuoka H. (2006). Failure mechanism in an extremely slow rock slide at Bitchu-Matsuyama castle site (Japan). Landslides.

[6] Damiano E., Avolio B., Minardo A., Olivares L., Picarelli L., Zeni L. (2017). A laboratory study on the use of optical fibers for early detection of pre-failure slope movements in shallow granular soil deposits. Geotech. Test. J..

[7] Pei H., Cui P., Yin J., Zhu H., Chen X., Pei L., Xu D. (2011). Monitoring and warning of landslides and debris flows using an optical fiber sensor technology. J. Mt. Sci..

[8] Chae B.G., Kim M.I. (2012). Suggestion of a method for landslide early warning using the change in the volumetric water content gradient due to rainfall infiltration. Environ. Earth Sci..

[9] Ponziani F., Pandolfo C., Stelluti M., Berni N., Brocca L., Moramarco T. (2012). Assessment of rainfall thresholds and soil moisture modeling for operational hydrogeological risk prevention in the Umbria region (central Italy). Landslides.

[10] Wu L.Z., Huang R.Q., Xu Q., Zhang L.M., Li H.L. (2015). Analysis of physical testing of rainfall-induced soil slope failures. Environ. Earth Sci..

[11] Martinović K., Gavin K., Reale C., Mangan C. (2018). Rainfall thresholds as a landslide indicator for engineered slopes on the Irish Rail network. Geomorphology.

[12] Baum L.B., Godt J.W. (2010). Early warning of rainfall-induced shallow landslides and debris flows in the USA. Landslides.

[13] Dixon N., Smith A., Spriggs M., Ridley A., Meldrum P., Haslam E. (2015). Stability monitoring of a rail slope using acoustic emission. Proc. Inst. Civ. Eng. Geotech..

[14] Smith A., Dixon N., Meldrum P., Haslam E., Chambers J. (2014). Acoustic emission monitoring of a soil slope: Comparisons with continuous deformation measurements. Géotech. Lett..

[15] Dixon N., Spriggs M.P., Smith A., Meldrum P., Haslam E. (2015). Quantification of reactivated landslide behaviour using acoustic emission monitoring. Landslides.

[16] Codeglia D., Dixon N., Fowmes G.J., Marcato G. (2016). Analysis of acoustic emission patterns for monitoring of rock slope deformation mechanisms. Eng. Geol..

[17] Naudetb V., Lazzari M., Perrone A., Loperte A., Piscitelli S., Lapenna V. (2008). Integrated geophysical and geomorphological approach to investigate the snowmelt-triggered landslide of Bosco Piccolo village (Basilicata, southern Italy). Eng. Geol..

[18] Chambers J.E., Meldrum P.I., Gunn D.A., Wilkinson P.B., Kuras O., Weller A.L., Ogilvy R.D. Hydrogeophysical monitoring of landslide processes using automated time-lapse electrical resistivity tomography (ALERT). Proceedings of the Near Surface 2009—15th European Meeting of Environmental and Engineering Geophysics.

[19] Chen Y.L., Uchimura T., Irfan M., Huang D., Xie J.R. (2017). Detection of water infiltration and deformation of unsaturated soils by elastic wave velocity. Landslides.

[20] Chen Y.L., Irfan M., Uchimura T., Nie W., Cheng G.W. (2018). Elastic wave velocity monitoring as an emerging technique for rainfall-induced landslide prediction. Landslides.

[21] Chen Y.L. (2016). Changes in Elastic Wave Velocity in a Slope Due to Water Infiltration and Deformation. Ph.D. Thesis.

[22] Orense R.P. (2004). Slope failures triggered by heavy rainfall. Philipp. Eng. J..

[23] Irfan M., Uchimura T., Chen Y.L. (2017). Effects of soil deformation and saturation on elastic wave velocities in relation to prediction of rain-induced landslides. Eng. Geol..

[24] Boyle A., Wilkinson P.B., Chambers J.E., Meldrum P.I., Uhlemann S., Adler A. (2018). Jointly reconstructing ground motion and resistivity for ERT-based slope stability monitoring. Geophys. J. Int..

